# A Comprehensive Review with Updated Future Perspectives on the Ethnomedicinal and Pharmacological Aspects of *Moringa oleifera*

**DOI:** 10.3390/molecules27185765

**Published:** 2022-09-06

**Authors:** Ummi Kalthum Azlan, Ahmed Mediani, Emelda Rosseleena Rohani, Xiaohui Tong, Rongchun Han, Norazlan Mohmad Misnan, Faidruz Azura Jam, Hamidun Bunawan, Murni Nazira Sarian, Hamizah Shahirah Hamezah

**Affiliations:** 1 Institute of Systems Biology, Universiti Kebangsaan Malaysia, UKM, Bangi 43600, Selangor, Malaysia; 2 School of Life Sciences, Anhui University of Chinese Medicine, Hefei 230012, China; 3 School of Pharmacy, Anhui University of Chinese Medicine, Hefei 230012, China; 4 Herbal Medicine Research Centre, Institute for Medical Research, National Institutes of Health, Shah Alam 40170, Selangor, Malaysia; 5 Faculty of Medicine, Manipal University College Malaysia (MUCM), Jalan Padang Jambu, Bukit Baru 75150, Malacca, Malaysia

**Keywords:** *M. oleifera*, bioactive compounds, polyphenols, pharmacological properties, nutritional composition, antioxidant activity

## Abstract

*Moringa oleifera* is an ancient remedy plant, known as the miraculous plant due to its many prominent uses and significant health benefits. It is a nutrient-rich plant, with exceptional bioactive compounds, such as polyphenols that possess several medicinal properties. Many significant studies have been carried out to evaluate the ethnomedicinal and pharmacological properties of *M. oleifera* in various applications. Therefore, this comprehensive review compiles and summarizes important findings from recent studies on the potential properties of different parts of *M. oleifera*. The pharmacological properties of *M. oleifera* have been studied for various potential biological properties, such as cardio-protective, anti-oxidative, antiviral, antibacterial, anti-diabetic and anti-carcinogenic effects. Therefore, the potential of this plant is even more anticipated. This review also highlights the safety and toxicity effects of *M. oleifera* treatment at various doses, including in vitro, in vivo and clinical trials from human studies.

## 1. Introduction

Plant-based products in medical research are currently one of the significant initiatives utilizing the potential properties carried by the bioactive compounds naturally found in plants. Many works have been carried out to incorporate plant products into safe drugs by synthetic strategies as well as to incorporate their potential effect into a regular diet. The *Moringa oleifera* plant, commonly known as ‘pokok kelor’ in Malaysia (natively known as drumstick tree or horseradish tree), is a plant that belongs to the *Moringaceae* family. It contains a great number of bioactive compounds, providing the pharmacological properties of the plant extract and contributing to the beneficial effects in humans [1,2,3].

Perpetually, this plant is a good source of naturally acquired medical benefits as most of them carry functional bioactive compounds, such as polyphenols and carotenoids [4]. Many studies have been carried out to investigate the medical significance of the bioactive compounds, possessing several biological activities such as antimicrobe, anti-inflammation and antioxidant. On top of that, the establishment of an optimized extraction method was also a game changer as the functional compounds can be extracted while keeping the original composition and structure. This has thus shown the authentic compounds to be better agents replacing synthetic compounds that are commonly toxic and have more carcinogenic effects.

**Figure 1:**
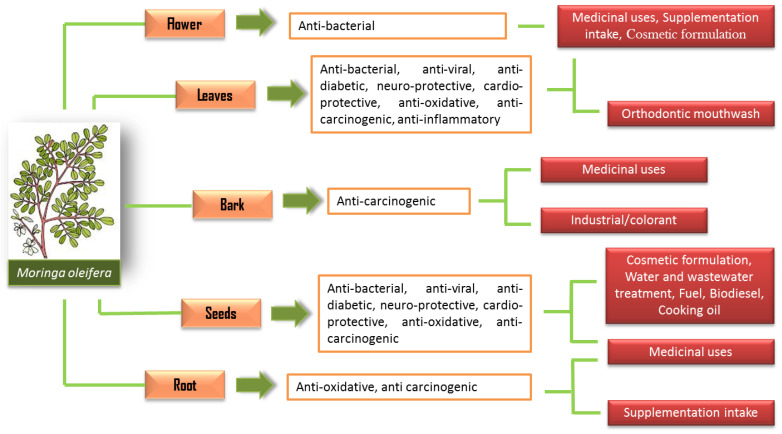
The uses of *M. oleifera* in various applications.

Yet, *M. oleifera* have been practically and traditionally used for many purposes such as traditional remedies for many diseases, food consumption and cosmetic value preparation even long before its nutritional and potential medical properties were discovered [5,6]. Since the 1970s, many significant studies have been carried out with remarkable findings that show the *M. oleifera* plant to be a nutrient-rich plant, with an exceptional combination of nutrients, amino acids and many more properties that are valuable medically [6]. Thus, the use of *M. oleifera* has been applied extensively in many applications following its potential properties (Figure 1).

Each part of the *M. oleifera* carries its own benefits, and the most widely studied parts are the leaves and seeds. The high polyphenolic contents in *M. oleifera* have been suggested to be one of the significant contributory factors to its beneficial effects on health. For instance, a study has reported that the ethanolic extract of *M. oleifera* leaves has been characterized by a high content of flavonoid constituents, such as isoquercetin, quercetin and kaempferol [7]. These compounds contribute to many of its pharmacological properties [8]. The bioactive compounds of *M. oleifera* have presented many remarkable medicinal properties with various potential biological activities. Therefore, this review encompasses previous studies on the effects of different parts of *M. oleifera* and its pharmacological properties. 

## 2. Methods of Literature Search

This review conducted an in-depth literature search of pre-existing data and research works published by compelling outstanding scientific journals, established online databases, related books, and book chapters from sources such as Web of Science (WoS), Google Scholar, Scopus and ScienceDirect. The literature search was performed to gather, evaluate, and summarize important findings from recent studies, in conjunction with older studies, to establish the findings on various topics related to the *M. oleifera* plant. This review on recent studies is focused on the period from 2014 to 2022; however, earlier references from previous studies were also included for a more comprehensive review.

## 3. Toxicology and Safety

The toxicity and safety assessment of *M. oleifera* has been described in many studies, and to date, to the best of our knowledge, there are no adverse effects of its consumption based on human studies that have ever been reported. Likewise, the potential toxicity of the plant has been assessed in both in vitro and in vivo studies (Table 1). Most of the in vitro studies involved the use of normal human cell lines and cancerous cell lines as the indices for the safety and toxicity of the *M. oleifera* extract treatment as summarized in Table 1.

In an animal study, the experimental rats were given an oral treatment of an aqueous leaf extract at 400, 800, 1600 and 2000 mg/kg doses. The study found that the administration of the highest dose (2000 mg/kg) was safe, and no fatality was observed, except for a decrease in body weight in a dose-dependent manner for the rats treated in a 21-day daily treatment cycle [15]. In another study, the experimental rats were given a treatment with 1000 and 3000 mg/kg of the leaf extract; genotoxicity was observed in rats treated with a 3000 mg/kg dose of the extract and not the lower dose [9]. However, even 1000 mg/kg of the extracts is still an excessive dose for normal application [20]. A cytotoxicity assessment of an aqueous seed extract of *M. oleifera* has found that even at the 2000 mg/kg dose of administration in mice, no systemic toxicity was observed with no significant changes in erythrocytes, platelets, hemoglobin, and hematocrit observed for the control group [10].

The evaluation for acute toxicity and LD_50_ of 70% ethanolic *M. oleifera* leaf extracts in white albino rats and rabbits has also been conducted [16]. The animals were continuously injected intraperitoneally with 150 mg/mL of the extracts every 5 min interval until mortality was reached. The results show that the lethal dose for acute toxicity was 6616.67 mg/kg for the rats and 26,043.67 mg/kg for rabbits. However, it was also observed that albino rats injected with 14 mL of a concentrated dose over 10 min showed no fatality, suggesting that water intoxication or accumulation of excessive fluid is likely the cause of fatality instead of the extract’s toxicity. This has also been confirmed by histopathology results, and it was concluded that ethanolic extracts of *M. oleifera* leaves have minimum toxicity and are not harmful to the animals if given within appropriate doses and ranges of time [16].

An acute oral toxicity study of the aqueous-methanolic leaf extract on female rats (Wistar albino) was performed, following OECD Guideline 425 with slight modifications [17]. Treated rats were fed orally with a 2000 mg/kg dose of the extracts, and blood was collected for biochemical analysis to evaluate important biomarkers of liver dysfunction. The study found that, at a 2000 mg/kg oral dose, the levels of aspartate aminotransferase (AST) increased significantly (*p* < 0.05), while non-significant increases (*p* > 0.05) were observed in the total bilirubin and a non-significant decrease (*p* > 0.05) in the levels of alanine aminotransferase (ALT) as compared to the control group. In addition, the postmortem analysis, which showed a non-significant increase (*p* > 0.05) in the hepatic index (liver to body weight ratio) and only mild distortions in the structure of liver cells via transverse liver section analysis, hypothetically concluded that the LD_50_ for the *M. oleifera* aqueous-methanolic leaf extract in female Wistar albino rats is >2000 mg/kg.

The toxicity profiles of crude methanol extracts of *M. oleifera* seeds and leaves and the relative impact on vital organs in Wistar rats were also evaluated through biochemical, hematological and histopathological analyses [18]. After 28 days of treatment with a daily oral feed of different doses (100, 200, 400 and 1000 mg/kg body weight) of the extracts, rats at all doses were observed with histopathological changes in the heart, liver, lungs, spleen and kidneys. At a 1000 mg/kg dose of seed extract treatment, a physical evaluation of agitation, confusion and disorientation was also observed but soon faded with no mortality. Other indices that were evaluated include white blood cells and platelet level, AST, ALT and alkaline phosphatase (ALP). This study observed that both seed and leaf extracts are safe for consumption in appropriate doses with enhanced immunity and offer hepatoprotective potential [18]. However, comparatively, the seed extracts showed more potential in a long-term application as more significant changes were observed in the indices.

In addition, an acute toxicity study has been conducted on *M. oleifera* leaf powder in Sprague Dawley rats with the toxicity classification method as described in the OECD Guideline 423 [19]. The experimental rats were treated with up to 2000 mg/kg of the dried leaf powder, and during the 14-day observation period, no adverse effect was observed for all groups, neither in the clinical signs nor gross pathology observation. The study indicated that the oral toxicity (LD_50_) of the dried leaf powder transcends beyond 2000 mg/kg doses, supporting findings in previous studies [15,17,19,21].

## 4. Origin, Geographical Distribution, Plant Description and Growth Condition of *M. oleifera*

**Figure 2:**
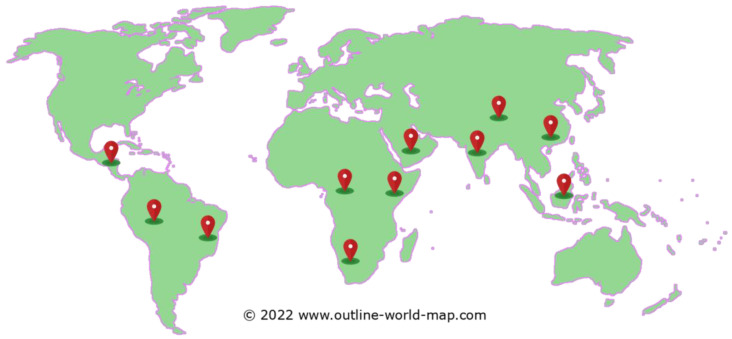
World map distributions of *M. oleifera*. World map image was obtained and modified from Outline World Map (free access).

*M. oleifera* is native to South Asia and is originally found in northeast Pakistan to northern West Bengal and India [5,6]. However, as its nutritional potential has been massively studied, it has been introduced to many parts of the world and is currently present and grows in almost all tropical countries (Figure 2). This has been possible as the growing environment for *M. oleifera* is atypical, where it can grow well in tropical and subtropical countries with conditions that are dry to moist tropical with annual precipitation of 760 to 2500 mm.

The optimum temperature for growing is between 18 and 28 °C, and it grows in any soil type, waterlogged and with heavy clay (pH between 4.5 and 8), at an altitude of up to 2000 m [6,22,23]. As the *M. oleifera* plant is edible, including almost all parts of the tree (leaves, roots, fruits, flowers and nutritious pod), it has been well spread and utilized in most countries [7,24]. The overwhelming discovery of its properties and potential has even earned it the name ‘nature’s gift’ or ‘miracle tree’. There are 13 species in the family that are fairly distributed all around Indian subcontinent origin. Owing to its Ayurveda reputation, *M. oleifera* is the most well-known species due to its rich nutrients and health benefits. As much as 1.1 to 1.3 million tons of the plants were produced by India alone on a yearly basis, in an area capacity of 38,000 ha of crop pods [25].

**Figure 3:**
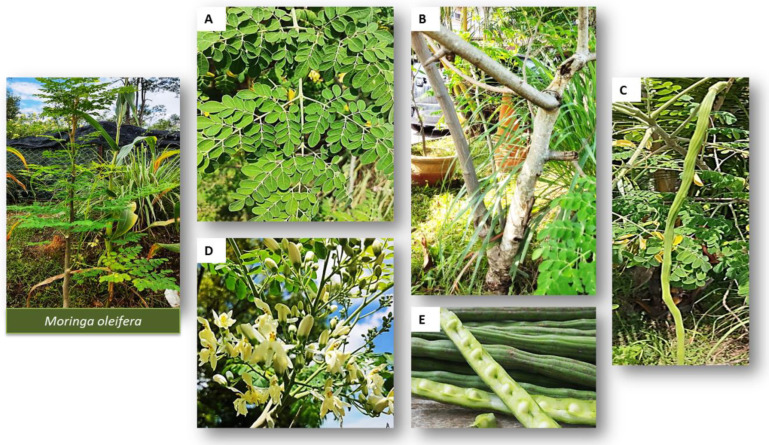
The different parts of *M. oleifera*: (**A**) leaves, (**B**) stem and bark, (**C**) pods, (**D**) flowers and sepals and (**E**) seeds.

A botanical description of the *M. oleifera* plant has reported that the tree can usually reach a height of 10–15 m with an approximate diameter of 45 cm and an abnormal-looking bole that is forked from the roots. The bark of the trees is smooth and fair in texture with toned-down shading and pale yellow color. The crown is often described as umbrella shaped with a spread opening with twigs and shoots that are short and bristly. The leaves can grow up to 90 cm long in alternate and opposite pinnae in inverse sets 5 cm up from the stalk of softwood [26]. Moreover, the flower of *M. oleifera* also has aromatic fragrance and five uneven petals that are a little longer that the sepals (Figure 3).

## 5. Phytochemical Properties of *M. oleifera*

*M. oleifera* has been reported to carry enormous phytochemical constituents that are beneficial and significant medically, which are mostly found on the leaves and seeds [6,20,27]. The leaves are found to be rich in many nutritious and bioactive compounds such as potassium, calcium, phosphorous, iron, protein, vitamins, carotenoids, polyphenols, isothiocyanates, tannins and more [7,28,29,30]. These bioactive compounds are accountable for many medicinal properties such as hypotensive, anti-cancer, antioxidant, antibiotic, antiulcer and anti-inflammatory properties, protection against signs of aging, malaria, typhoid fever, diarrhea and dysentery and colitis hepatoprotection [7,31,32,33].

**Figure 4:**
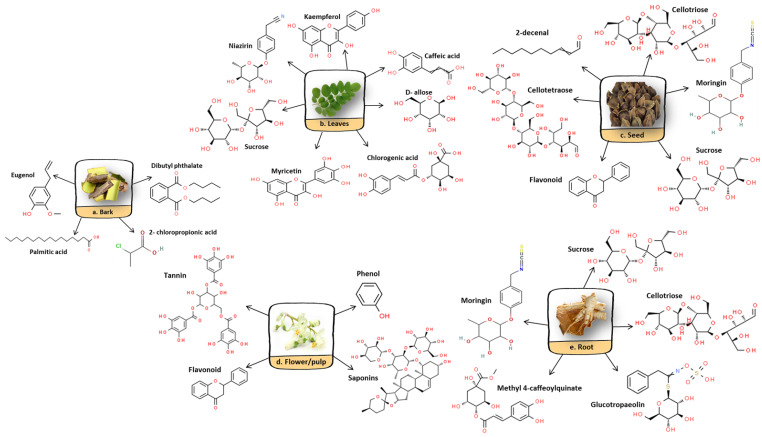
Bioactive compounds present in different parts of *M. oleifera*.

From the many bioactive compounds, dried *M. oleifera* leaves are good sources of polyphenolic compounds where the principal compounds are flavonoids and phenolic acids [5,7]. The concentration of polyphenolic compounds in *M. oleifera* leaves was observed to reach higher than in certain fruits and vegetables with an approximate range from 2090 to 12,200 mgGAE/100 g of dry weight (DW) or 1600 to 3400 mgTAE/100 g of DW (differs according to the extraction protocol). Phenolic acids and flavonoid compounds help to eliminate oxygen free radicals from the body which improves cardiovascular and cerebrovascular diseases and enhances antioxidant activities as well as anti-tumor properties [34,35]. The phenolic acids extracted from *M. oleifera* leaves are mainly gallic, chlorogenic, ellagic and ferulic acids, while the flavonoid contents are mostly quercetin and kaempferol. In addition to that, other parts of *M. oleifera* also contain bioactive compounds that are attributable to each of the potential pharmacological properties and benefits (Figure 4). 

## 6. Absorption, Metabolism and Excretion

*M. oleifera* has been regarded as the miraculous tree aptly called the ‘tree of life’ due to its immense nutritional benefits. Thus, the bioavailability of the plant has been studied as one of the crucial factors in utilizing its nutritional benefits. Despite having a worthy content of iron, several studies have found that *M. oleifera* plants have low iron bioavailability [36,37]. The high polyphenolic contents of *M. oleifera* plants may exhibit a conflicting inhibitory effect on iron absorption via the formation of non-bioavailable polyphenol–iron complexes [37,38,39]. However, the formation of inhibitory complexes that lead to poor iron absorption into the body relies specifically on the structures of polyphenol compounds [40,41]. In addition, a study has suggested that the low iron bioavailability is caused by the presence of high phytic acid content in the *M. oleifera* sample, and the removal of phytic acid during its processing may improve the bioavailability [36]. 

In addition, *M. oleifera* is rich in minerals and vitamins. Similar to the iron bioavailability, dried *M. oleifera* leaves contain a profuse calcium content, but the presence of a substantial amount of oxalic acid in the leaves has caused interference in calcium absorption [42]. In a comparative study, where experimental rats were nourished with a calcium-rich diet from *M. oleifera* leaves and a milk diet, the milk diet was observed to have better absorption and calcium retention as compared to the *M. oleifera* diet. Despite having the same calcium content, *M. oleifera* leaves were found to contain oxalate, which hypothetically suggested the cause of reduced calcium bioavailability. However, as much as 73% of calcium from the *M. oleifera* diet was still absorbed, with 59% of it being retained, proposing the *M. oleifera* diet as an adequate alternative source in the case of milk deprivation [42,43]. The conflicting bioavailability effects caused by the presence of phytic and oxalic acid in dietary consumption have ironically labeled them as anti-nutritional factors [44].

In addition to that, vitamins A and B are among the significant reported nutrients of *M. oleifera* leaves and one of the most abundant natural sources for β-carotene and provitamin A carotenoid [45,46]. In vivo and in vitro studies found that the natural vitamin sources of *M. oleifera* are highly bioavailable [47,48]. In the in vivo study, it was observed that not only did the *M. oleifera*-fed rats have a splendid food intake and weight gain, but the levels of vitamin A observed were also significantly higher than in the control group. This indicated that the rats fed with the *M. oleifera* diet had good absorption of nutritional values. 

Other than that, the nutritional benefits of *M. oleifera* include all essential amino acids that act as the building blocks for proteins necessary for body nourishment. A randomized control trial observing the impact of *M. oleifera* consumption on lactating women carried out in Senegal found that the body mass index (BMI) of lactating women fed with the *M. oleifera* diet remained stable as compared to the control group. The reduced weight loss in the *M. oleifera*-fed group suggested an efficient absorption of a high protein content [37]. Other studies have also found that most of the amino acids or proteins in *M. oleifera* are highly digestible which equates to its bioavailability [42,49].

## 7. Pharmacological Properties of *M. oleifera*

**Figure 5:**
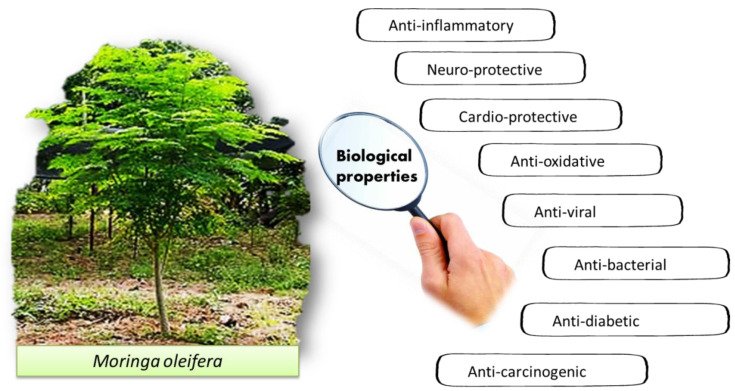
The reported biological properties of *M. oleifera*.

The pharmacological properties of *M. oleifera* have been studied for various potential biological properties, such as cardio-protective, anti-oxidative, antiviral, antibacterial, anti-diabetic and anti-carcinogenic effects (Figure 5). Table 2 shows the comprehensive review of many research findings that includes the extracts of different parts of the plant, the extraction solvent, the drying and extraction method, the analytical approach, bioassays, the findings and the respective pharmacological properties.

### 7.1. Anti-Oxidative Effects of M. oleifera

The high antioxidant activities of *M. oleifera* are often associated with its high content of polyphenol compounds. Antioxidant activities can be beneficial in many applications, and much evidence has shown that dietary polyphenols help to alleviate the complications of many critical illnesses, such as cancer, cardiac diseases and chronic inflammation that are commonly related to oxidative stress. Mechanistically, polyphenol compounds are secondary metabolites in plants known to be potent antioxidant agents that complement and add value to the activities of antioxidant vitamins and enzymes against oxidative stress [87]. They can neutralize free radicals by donating an electron or hydrogen atom dubbed as the main contributor to overall antioxidants in fruits, followed by vitamins [88]. However, studies found that chlorophyll has higher radical scavenger and reducing agent potential than phenolic compounds and flavonoids [89,90].

The nephroprotective and antioxidant effects of *M. oleifera* were appraised in paracetamol-induced nephrotoxic albino rabbits [79]. The study used the *M. oleifera* seed powder for oral administration in the treatment group at different doses (200, 400 and 600 mg/kg). That study found that, at an optimum dose of 600 mg/kg, the seed-powder-treated group demonstrated an alleviated damaging effect of paracetamol-induced renal damage in the rabbit. The authors stipulated that the alleviated damage was due to an altered lipid peroxidation process, and this may suggest the promising potential of *M. oleifera* in the treatment of renal failure or as an alternative to enhance the therapeutic value of the nephrotoxic agent [79]. 

The antioxidant effect of Japanese *M. oleifera* products, which consist of the herbal leaf tea and stem, have been investigated via free radical assays that target superoxide anion (O_2_^−^) radical generation systems [52]. This study used Trolox as the control standard for the determination of free radical scavenging capacity in the sample as it is an analog of α-tocopherol which is water soluble. Results show that the hot extracts of *Moringa* teas have lower scavenging activities than the Trolox standard in the tested synthetic free radical models [52]. However, the extracts also demonstrated an elevated O_2_^−^ radical scavenging activity than Trolox in the phenazine methosulfate–NADH–nitroblue tetrazolium and xanthine oxidase assay systems. Other than that, the tea extracts potently suppressed the cellular O_2_^−^ radical generation in incubated human neutrophils as compared to the Trolox standard. It was stipulated that, among the polyphenol contents of *M. oleifera*, caffeic acid and chlorogenic acid are the two compounds that are crucial for O_2_^−^ specific radical scavenging capacity that is stronger than Trolox. Thus, it was suggested that the tea extracts consisting of leaves and stem parts of *Moringa* are a good alternative for natural antioxidants that help prevent O_2_^−^ radical mediated conditions.

The antioxidant activity of the *M. oleifera* leaf has been investigated across the age of the leaf (30, 45 and 60 days) and extraction solvent (methanol, ethanol and aqueous) by using radical scavenging assays such as DPPH, 2, 2′-Azino-Bis-3-Ethylbenzothiazoline-6-Sulfonic (ABTS) and anti-peroxide activity (APA) [91]. The TPC, TFC and chlorophyll content of the leaves was determined as part of the correlation in the study. The study found that total phenolic (TPC) and flavonoid (TFC) content was increased with age as the highest readings were observed at 60 days of leaf maturation in ethanol and methanol solvents but peaked at 45 days for the aqueous extract. The highest TPC was observed for the methanolic extract while the highest TFC was observed in the ethanolic extract. On the other hand, the ethanol and methanol extracts were observed to have similar chlorophyll contents that were significantly higher than in the aqueous extract. However, the chlorophyll content remained constant or reduced after it peaked at 30 days for all three solvents. Ethanolic leaf extracts showed the highest DPPH activity, while both ethanolic and methanolic extracts demonstrated similar ABTS+ activity. However, the authors also proposed that chlorophyll is the main contributor to antioxidant activities as there is evidence of a positive correlation between chlorophyll content and DPPH, ABTS and APA. The study concluded that ethanolic and methanolic extracts showed higher antioxidant activity than the leaf aqueous extract and 45 days of age is the optimum condition for extraction with the highest antioxidant potential.

Aside from that, the phytochemical content and antioxidant activity for different types of extraction of *M. oleifera* seed kernels (methanol, acetone and water) originating from Bangladesh were also appraised in a previous study [81]. In addition to the TPC, TFC and total tannin content evaluation, the in vitro antioxidant activities were determined by performing DPPH, ABTS, NO (nitric oxide) free radical scavenging and FRAP assays [81]. It was observed that the aqueous extracts showed the highest activities for scavenging DPPH, ABTS and NO free radicals as well as significant free radical scavenging activities and reducing power, higher TPC and TFC than the methanol and acetone extracts. This is in contrast with the previous finding as the aqueous extract was dubbed the most potent natural antioxidant agent [91]. However, the author also suggested that as future research, the study of the isolation of active compounds from the extracts may elucidate more rationale on the current finding. 

In another locality, a study has been conducted to systematically evaluate the anti-inflammatory and antioxidant activities of ethanolic extracts of the leaves, seeds and roots of *M. oleifera* harvested in Kenya [62]. It was demonstrated that the leaf extracts showed the highest DPPH and FRAP activities while the leaf and root extracts displayed potential ABTS radical scavenging activities [62]. In addition, the leaf and seed extracts exhibited anti-inflammatory activities by the suppression of NO production. Phytochemical analysis via HPLC-UV/ESI-MS/MS found that the leaves of *M. oleifera* contain substantial amounts of flavonoid and phenolic acids as compared to the seed and root parts. As the positive correlation analysis found that flavonoid content is directly associated with antioxidant and anti-inflammatory activities, the high TPC and TFC of the leaves thus suggest it is a more potent source of anti-inflammatory and antioxidant activities as compared to other parts of the plant.

### 7.2. Antiviral Effects of M. oleifera

*M. oleifera* has been vastly studied as a potent antiviral agent. Years before the establishment of vaccine development and advancement, *Moringa* plants were traditionally used to treat many viral infections such as smallpox and chickenpox. Even though it has never been scientifically proven that *M. oleifera* plants are effective against viral infection due to the inability to conduct extensive research as the World Health Organization (WHO) has declared the world free of smallpox infection since May 1980, the potential of the plants as promising antiviral agents against other viral infection has continually been studied [92,93]. Following the reports of many authors, *M. oleifera* extracts exhibited potent inhibitory activities against many viral infections such as the influenza A virus (IAV) [94], Herpes simplex virus type 1 (HSV-1) [95,96,97], foot and mouth disease virus (FMDV) [63,98], hepatitis B virus (HBV) [99,100], human immunodeficiency virus (HIV) [11,101], Epstein–Barr virus (EBV) [102] and Newcastle disease virus (NDV) [103,104].

A study has demonstrated that Moringa A, the new compound from the *M. oleifera* seed, is effective against IAVs as it impedes the replication of the virus and protects the host cells from the cytopathic effect [94]. It was also found in the in vitro study that the compound can disrupt the cellular protein transcription factor EB (TFEB) and in turn decelerate autophagy in infected cells [94]. The *M. oleifera* leaf extract has also been found to be effective in an in vitro study against FMDV at 100 µg/mL, and it is toxic to the cells at a concentration of 200 µg/mL and higher [63,98]. It has been proposed that one of the thiocarbamate compounds, namely the niaziminin found in *M. oleifera* leaves, exhibits antiviral activities against the FMDV. This niaziminin compound has been discovered before against EBV, where the reaction of the compound and 4-[(4′-O-acetyl-alpha-L-rhamnose loxy) benzyl] isothiocyanate inhibited the activation of EBV [102].

From the survey, it was found that the extracts of *M. oleifera* are often consumed as part of a supplementation diet as part of an alternative to consuming conventional medicine. Even though research on the risk of the herb–drugs interaction is still scarce, no adverse effect has ever been reported. As there are many claims suggesting the ability of *Moringa* to improve the quality of life of people living with HIV/AIDS (PLWHA), a study has been carried out to investigate the in vitro inhibitory activities of *M. oleifera* extracts on lentiviral vector infectivity [11]. Results show that all ethanolic, methanolic and water extracts of *M. oleifera* were active against the HIV-1 lentiviral vector, and the early event of viral replication was inhibited. The potential of *M. oleifera* extracts in the selective inhibition of viral replication has suggested that they could serve as potent antiretroviral lead molecules.

In addition, considering the recent COVID-19 outbreak that has now become a pandemic, there has also been an attempt to investigate the potential of *M. oleifera* as a supplementary diet in enhancing the immune system. A review has suggested that *M. oleifera* can be effective against COVID-19 in a comprehensive way such that the plant acts as an immune booster and may increase the survival rate of people with SARS-CoV-2 infection [105]. There are many bioactive compounds of *M. oleifera* that show promising potential against COVID-19 infection such as kaempferol, quercetin, morphine, pterygospermin and apigenin-7-*O*-rutinoside [105,106,107]. Among all, the apigenin compound showed the highest activity against SARS-CoV-2- MPro, one of the main proteases of COVID-19, further concluding the potential of the *Moringa* plant as an immune booster against SARS- CoV-2 (COVID-19).

### 7.3. Antibacterial Effects of M. oleifera

The bacterial species that have been tested against the potent *M. oleifera* include water-borne pathogens, diarrhea-causing bacteria, drug-resistant bacteria and many more. A study has observed that the hexane and methanol seed extract of the plants exhibited inhibition of water-borne pathogens such as *Salmonella typhii*, *Vibrio cholera* and *Escherichia coli* [86]. Therefore, it was proposed that the antibacterial effect of *M. oleifera* could serve as a natural antibacterial agent in managing bacteria-caused water-borne diseases. Another study has also been carried out to investigate the antibacterial properties of different parts of *M. oleifera* in an approach to create natural dental care from the plant. Among the many attempts to formulate the right extracts into an experimental toothpaste and mouthwash, an ethanol extract of the leaves showed the highest antibacterial activities against *S. aureus* and *Streptococcus* mutant growth, with the experimental toothpaste exhibiting higher activities than the mouthwash [64]. The vast application of antibacterial agents derived from natural products has been crucial as it is more environmentally friendly, less toxic and a cheap and sustainable method for disease management and to improve the quality of life, especially in rural and developing countries.

In one study, both ethanol and methanol extracts of *M. oleifera* leaves showed a significantly higher (*p* < 0.05) inhibitory effect at a higher concentration of 120 mg/mL as compared to an aqueous extract against *E. coli*, *S. aureus* and *Pseudomonas aeruginosa* [53]. The finding suggests that the antibacterial activity of *Moringa* leaves is effective against both Gram-positive bacteria (*S. aureus*) and Gram-negative bacteria (*E. coli* and *P. aeruginosa*). In another study, an *M. oleifera* leaf extract was also tested against isolated multidrug-resistant (MDR) *E. coli*, *S. aureus* and *P. aeruginosa* by using the agar disc diffusion method. The results show that the chloroform extract had the highest antibacterial activity (9.32 ± 1.45 mm), while the aqueous extract had the lowest activity (0.27 ± 0.27 mm) [108]. The antibacterial activity observed against MDR bacteria added value to *M. oleifera* as a promising treatment alternative for infections caused by MDR bacteria. 

The antibacterial effect of *M. oleifera* is the most anticipated property due to the massive application of antibacterial agents in various settings. *M. oleifera* is astonishing as a plant because every part of it, which includes the seed, root, bark, stem and leaf, has been described to harbor its own potential, coupled with the best extraction method and solvents that established its potency. Table 3 describes more studies from different authors that have investigated the antibacterial properties of *M. oleifera* against various species in many applications.

### 7.4. Anti-Diabetic Effects of M. oleifera

Diabetes mellitus (DM) is a metabolic disease that causes high blood glucose due to the body’s inability to produce sufficient or functional hormone insulin to regulate blood glucose. Many studies have demonstrated the potential of *M. oleifera* as anti-diabetic agents for the treatment of this metabolic disease due to the high presence of polyphenols that help to reduce blood glucose [109] and improve sexual dysfunction [110]. The leaf powder of *M. oleifera* was found to have quercetin-3-glucoside and fibers that give mitigating effects on glucose intolerance [39]. In addition, the leaves are also rich in unique Moringa isothiocyanate (MIC) compounds that possess high biological activities and evidence of therapeutically active constituents [111]. It is also evidently suggested that a potential wound dressing formulation containing extracts of *M. oleifera* may help with wound management that potentially aggravates diabetic conditions [112].

The oral administration of ethanolic extract of *M. oleifera* leaves has been investigated for the anti-diabetic and liver function indices in Alloxan-induced diabetic rats via glucometer and spectrophotometric methods [66]. The results show that there is a significant decline in the glucose level of the treated rats and elevated levels of liver indices ALT, AST and ALP in a dose-dependent manner. It was also demonstrated that the levels of albumin and bilirubin changed according to doses; for example, a 200 mg/kg dose showed an increase in the albumin level, but at higher doses, the albumin levels were reduced. It can be stipulated that aside from providing anti-diabetic potential, the extracts also help to protect from liver damage, and 400 mg/kg was observed to be the safest dose. 

In another study, the aqueous ethanolic extract (95, 75, 50, 25% *v*/*v* and 100% water) of *M. oleifera* leaves was fed orally to experimental rats to investigate the hypoglycemic activities and contribution to intraperitoneal glucose tolerance test (IPGTT) data [67]. As a 95% (*v*/*v*) ethanolic extract (at 1000 mg/kg) showed the highest activities, it was submitted for liquid-to-liquid fractionation into hexane, chloroform, ethyl acetate, butanol and water for more screening of potent anti-diabetic activities [67]. Among all extracts and fractions, the 95% ethanolic extract and only butanol fraction showed an effect by alleviating blood glucose concentration after administration to diabetic rats. However, no hyperglycemic effect was observed in normal rats. The TLC and HPLC analysis determined the presence of quercetin 3-β-D-glucoside, kaempferol-3-*O*-glucoside and crypto chlorogenic acid in the extracts that stipulated antihyperglycemic potential. Thus, the authors suggested the potential of the extract and fraction as alternative treatments for diabetes and recommended further investigation for drug discovery.

Aside from the leaves, the seed extracts of *M. oleifera* have also been studied for their potential as anti-diabetics. There was a potential of the aqueous extract and oil of *M. oleifera* seeds against several biochemical markers in streptozotocin-induced diabetes mellitus albino rats [80]. The serum was collected for the determination of blood glucose, body weight, albumin, urea, creatinine, electrolytes (Na+, K+ and Cl^−^)- and the levels of enzyme markers for liver damage (AST and ALT). The results show that at 100 mg/kg and 200 mg/kg doses of aqueous extract treatment of diabetic rats, a significant reduction in serum glucose was observed [80]. Other than that, there was also a decrease in urea and creatinine levels that were significant as compared to the diabetic untreated group. In addition to that, the extract was also observed to ameliorate the hepatic function as low levels of enzyme markers were recorded in the treated group. Thus, this proposed the potential of the *M. oleifera* seed extract as an anti-diabetic with remarkable nephron-protective activity.

In a more recent study, the effect of the ethanolic leaf extract at two different doses (250 and 500 mg/kg) on the metabolic glucose, melatonin and lipid profile and liver and kidney function in Alloxan-induced diabetes was investigated [68]. After 60 days of oral treatment of the extracts, results showed that there was a significant decline in blood glucose, total cholesterol, triglycerides and the levels of low-density lipoprotein (LDL), ALP, ALT and AST. Elevated levels of serum melatonin, lactate dehydrogenase (LDH) and high-density lipoprotein (HDL) were also recorded in the diabetic group as compared to the control group. The authors thus stipulated that the ethanolic leaf extract treatment of diabetic rats helps to reinstitute the metabolic changes to normal levels.

### 7.5. Anti-Carcinogenic Effects of M. oleifera

The anti-carcinogenic potential of *M. oleifera* is one of its medical benefits that is worth investigating due to the high content of various phytochemicals, supported by much evidence of low toxicity that ensures the safe application of the plant [24,113,114]. *M. oleifera* is rich in phenolic acids and flavonoid compounds that are known for their potential as antioxidants and anti-cancer agents. As oxidative stress is one of the causative agents of cancer development, the presence of compounds harboring antioxidant properties may interfere with the floating free radicals and reduce oxidative stress. This will consequently help to prevent cancer. 

The anti-carcinogenic effect of different parts of *M. oleifera* (leaf, bark and seed extracts) against the MDA-MB-231 and HCT-8 cancer cell lines has been studied [69]. The study found that the leaf and bark extract showed significant anti-cancer effects as compared to the seed extract. The leaf- and bark-extract-treated cell lines showed low cell survival with a remarkable reduction in cell growth as well as cell motility. In addition, the apoptosis assays showed significant increments of apoptotic cells for the two extract groups. The GC-MS analyses demonstrated the presence of many targeted anti-cancer compounds such as eugenol, isopropyl isothiocyanate, D-allose and hexadeconoic acid ethyl ester that indicated the anti-cancer properties of *M. oleifera*. The authors claimed that the study was the first to report the anti-cancer potential of the bark. It was suggested that leaf and bark extracts of *M. oleifera* exhibited anti-cancer activity in both cell lines, and thus new potent agents can be proposed in the treatment of breast and colorectal cancers [69].

The potential of *M. oleifera* leaves has been further investigated in another study, against different cell lines of human hepatocellular carcinoma HepG2 cells [58]. Following the analysis of apoptotic signals, results show that the leaf extract triggers the apoptosis reaction in HepG2 cells. Moreover, the hollow fiber assay (HFA), using immunodeficient nude mice, demonstrated a notable reduction in both HepG2 cells and A549 non-small cell lung cancer cell proliferation after the oral administration of the leaf extract. It was proposed that the remarkable tumor inhibition activities may have resulted from the high bioactive compound content in the extracts, thus suggesting its potential as a promising anti-carcinogenic agent [58]. 

The ethanolic extract of *M. oleifera* has also been evaluated for its regulatory activity in leukemic Wistar rats via a tumor necrosis factor-α (TNF-α) assay [70]. The ethanolic extract was orally administered to the rats pre-, during and post-leukemia induction, in a compelling 8 weeks of total duration. The plasma sample was collected for the TNF-α analysis by using an enzyme-linked immunosorbent assay (ELISA). The level of TNF-α was the highest in the non-treated group, followed by the *M. oleifera*-treated group, and the lowest was observed in the control group. TNF-α is a known pro-inflammatory cytokine that is released upon the activation of macrophages or monocytes to mediate various cellular events such as the stimulation of other functional cytokines, cell proliferation, differentiation and apoptosis. One previous study has found that the reduction in the TNF-α level signifies the response against treatment while elevated TNF-α levels are indicative of the active disease progress [115]. Thus, the authors proposed that the TNF-α level may be a suitable indicator for the clinical efficacy of anti-cancer therapy.

The extract of *M. oleifera* seeds, roots, stems and leaves in different ethanol concentrations (50, 70 and 90%) was appraised for the antioxidant and anti-proliferative properties in different cell lines from a previously reviewed study, the head and neck cancer (HNC) cell lines, CNE-1 and CAL27 [71]. Prior to the investigation of the cell line, the TPC, TFC and antioxidant levels were determined for all the different extracts. The results of this study suggest that the aqueous leaf extract showed the highest antioxidant activities, but the 70% ethanolic extract recorded high antioxidant activity for the other parts of the plants (seeds, roots and stems). In addition to that, all the extracts showed notable anti-cancer activities in the tested cell line where the proliferations of HNC cells were impeded by the suspected apoptosis inducement. Interestingly, the stem extracts exhibited the strongest apoptotic induction, followed by the leaf extracts. This thus concluded that the *M. oleifera* extract possesses remarkable antioxidants and anti-proliferative potentials that may be helpful in the management and treatment of head and neck cancer.

### 7.6. Cardio-Protective Effects of M. oleifera

Cardiovascular abnormalities are one of the most concerning conditions ever existing medically, and all related complications have contributed to the high mortality throughout the world. The use of phytochemicals from natural medicinal plants has been extended to various applications including as a cardio-protective agent as more evidence from scientific research has suggested its potential. Bioactive compounds such as diosgenin, isoflavones, sulforaphane, carotinized, catechin and quercetin have been determined to contribute to cardio-protection and alleviating cardiac-related complications [116]. *M. oleifera* has been studied as potent medicinal plants for cardio-protection due to their abundant phytochemicals such as polyphenols that perform cardio-protection by impeding hepatic cholesterol and lipoprotein metabolism and mitigating the inflammatory response [117]. The ethanol and aqueous extracts were found to contribute significantly to reduced systolic and diastolic blood pressure in spontaneously hypertensive rats [118].

A study has evaluated the cardio-protective effect of the aqueous extract of *M. oleifera* leaves on Wistar albino rats via investigations of the lipid profile as well as the cardio-toxicity effect [51]. In this study, the rats were administered with potassium bromate to induce toxicity on the cardiac tissue, and then *Moringa* extracts were applied to investigate the detoxifying effect. Potassium bromate is a potent cardio-toxin that increases lipid peroxidation and reduces heart antioxidant activities. In the potassium bromate-induced rats only, cardiac dysfunction was indicated by the elevated cardiac biomarker enzymes AST, ALT, ALP and other tested components on cardiac tissues. Results show that the extract of *M. oleifera* demonstrated cardio-protection potential on the potassium bromate- induced cardiac oxidative damage in rats as the antioxidant loss was alleviated and the cardiac dysfunction was restored [51].

Other than that, the potential of *M. oleifera* seed powder has been evaluated in spontaneously hypertensive rats (SHRs) where the SHRs were given oral administration of food containing the seed powder, and the cardiac effects were determined [76]. Hypertension is a condition of perpetuated high blood pressure that may result in cardiac complications with an escalated risk of heart attack/heart failure. Upon oral treatment of *Moringa* seed powder, no changes were observed in the rats’ blood pressure, except for a decrease in nocturnal heart rate with ameliorated cardiac diastolic function. The authors also suggested that the seed powder treatment may have an effect on the signaling pathways associated with pressure-overload-induced left ventricular hypertrophy such as the calcium-regulated mechanism. However, an in-depth study is needed to elucidate the exact mechanism involved in the *Moringa* cardio-protective potential. 

A study has been conducted to investigate the effect of *M. oleifera* seed powder on the oxidative and nitrosative vascular stresses in SHRs [77]. Reduced vascular stresses were observed in the *Moringa*-treated SHR aortas, associated with a decline in the free 8-isoprostane circulating level, vascular p22phox and p47phox expressions and the upregulation of SOD2. After the treatment, it was found that there were decreased iNOS and NF-κB protein expressions, which resulted in reduced circulating nitrites and C-reactive proteins that are often elevated in normal SHRs. The study also found that the treated-SHR group showed an enhanced resistance of the arteries against the endothelium-dependent carbachol-induced relaxation functional test. This study presented an overall vascular antioxidant, anti-inflammatory and endothelial protective potential of *M. oleifera* seed powder in a supplementary diet against cardiovascular complications indicated by oxidative stress and inflammation [77]. 

In addition, the cardio-protective effect of the methanolic *M. oleifera* seed extract has been studied in isoproterenol-induced myocardial infarction (MI) in Wistar albino rats [85]. The treatment lasted for 32 days, the fasting blood samples were collected for the determination of serum cardiac biomarker enzymes and the lipid profile, while the heart tissue was collected for the evaluation of myocardial marker enzymes (LDH, CPK, AST, ALT and CK-MB) and antioxidant enzymes (GSH and LPO). The study found that the rats treated with the methanolic seed extracts showed a positive effect that reversed all the altered regulation of the tested biomarkers as compared to isoproterenol-induced rats. The positive reversed effect of *Moringa* was observed as follows: the isoproterenol caused a significant increment in serum myocardial enzymes (LDH, CPK, AST, ALT and CK-MB) and lipid profiling parameters, but the *Moringa*-treated group showed a decrease in the levels. The isoproterenol reduced the myocardial enzymes in the heart tissue significantly, but the *Moringa* pre-treated rats presented elevated biomarker enzyme levels in a dose-dependent manner. 

The *M. oleifera* seed has also been evaluated for its potential in ischemic heart diseases. The *M. oleifera* seed powder was orally administered to wild-type C57/BL6 male mice by feeding the mice a diet containing the seed powder. The study found that the *M. oleifera* treated group had a reduced MI-induced mortality and alleviated cardiac dysfunctions in MI mice [78]. In addition, it was observed that post 28 days of MI inducement in mice, there was a significant increment in ejection fraction and fractional shortening, with more data suggesting that the *Moringa* treatment attenuates MI-induced infarction size and cardiac remodeling. The study elucidated that the mechanistic role of *M. oleifera* seeds in ischemic heart diseases is indicated by the inhibition of MI-induced apoptosis and subdued cardiac fibrosis. The authors concluded that oral administration of *M. oleifera* seed powder potentially exhibits anti-apoptosis and antioxidant effects which are critical for mitigating MI damage to cardiac function in MI-induced mice model. 

## 8. Clinical Trials and Human Studies

Clinical trials are part of a research strategy that is performed on humans, essentially to investigate any behavioral, medical or surgical intervention of targeted properties. Following the enormous nutritional claims and research data from related scientific journals, clinical trials are needed mandatorily before the claims are accepted for massive application. For *M. oleifera*-related clinical trials, there are approximately 25 interventional studies that have been registered under the registry of clinical trials (clinicaltrials.gov) where 15 of them have been completed, and 10 are still ongoing. Among the trials, 17 of them are supplementation diets of *Moringa*, 6 drug interventions, and 2 are *Moringa*-based mouthwashes for orthodontic application. Table 4 shows the overview of all registered clinical trials of *M. oleifera*, obtained from the clinicaltrials.gov website. 

There are many human studies that have been carried out on evaluating the potential of *M. oleifera* in various conditions and applications. The effect of *M. oleifera* on the human lipid profile, blood pressure and body mass index involving 16 normal human subjects showed that, over the course of 15 days, all lipid profile components tested showed no significant (*p* > 0.05) changes in both diet groups (0.03 g/kg body weight and 0.07 g/kg body weight) [119]. It was also noted that there was a non-significant decrease in the blood pressure level and body mass index of normal human subjects. However, it was postulated that certain effects may be more prominent if higher doses were applied to obese human subjects, thus calling for future investigation [119]. 

Other than that, *M. oleifera* leaves were also tested for the effect on the hematological indices in human subjects [120]. The subjects were divided into low- (0.038 g/kg) and high-dose (0.077 g/kg) treatment groups over 14 days of treatment. The results show that there was an elevated platelet reading that is significant in the low-dose group as compared to the high-dose group. There were also non-significant increments in red blood cells and reduced white blood cell (WBC) levels in both groups. The authors concluded that the *M. oleifera* leaf contributed to hematopoietic potential in humans as there was an improved platelet and red blood cell count. On the contrary, with the decrease in WBC levels, the immune enhancement potential is still scarce. 

A study involving normoglycemic human volunteers aimed to investigate the anti-hyperglycemic effect of functional *Moringa* tea [121]. The tea was given to the subjects, and their blood glucose was measured for 150 min at 30 min intervals. It was observed that there was a suppression of the blood glucose level as compared to the negative group in a dose-dependent manner. The author suggested that *M. oleifera* can be beneficial as an anti-diabetic diet as it helps maintain blood glucose levels and prevent additional symptoms. The study reported that the low-dose group showed more anti-hyperglycemic effects by restricting glucose absorption at the intestinal level while the high-dose worked more in circulation [121]. However, extensive research needs to be carried out to understand more about the effect on diabetic patients and the exact mechanism that it incurs. In addition, another anti-diabetic study in human subjects was carried out with 10 healthy volunteers to evaluate the effect of *M. oleifera* on plasma glucose and the secretion of insulin [122]. The human subjects were given an oral dose of *M. oleifera* in several dosages (0, 1, 2 and 4 g), and the results show that, after the supplementation routine, there was elevated plasma insulin that was significant after the high-dose diet, but no differences were observed for plasma glucose. It was proposed that the high-dose *M. oleifera* leaf diet helps to enhance insulin secretion in healthy volunteers and is a potential agent for the treatment of type 2 diabetes. Thus, prospective studies are needed to evaluate the potential in type 2 diabetic patients, as well as to investigate the effect on insulin secretion/resistance. 

A randomized, double-blind, placebo-controlled parallel group intervention study has been conducted with subjects with prediabetes, applying the *M. oleifera* diet (2400 mg/day) concurrent with the placebo group for over 12 weeks of study [123]. The study found that there were significant differences observed between the test group and the placebo-controlled group which showed contradicting results. During the intervention, tested components such as fasting blood glucose (FBG) and glycated hemoglobin (HbA1c) showed a decreasing pattern in the *Moringa* tested group while increasing in the placebo group. However, in the tested scores for measured microbiota, hepatic and renal function markers or appetite-controlling hormones were not significant. It was concluded that the supplementation diet helps to provide a natural anti-hyperglycemic potential in humans as also supported in previous studies [121,123].

## 9. Conclusions and Future Perspectives

*M. oleifera* is an important and well-known plant in the field of Ayurveda since it has traditionally been utilized for a variety of purposes. It is famously known to have endless potential as a supplementation food and nutraceutical due to the presence of abundant phytochemical constituents. Aside from being a good source of a variety of useful bioactive chemicals, *M. oleifera* is also an anticipated plant in nutritional research as it has only a minimal need for crop growth requirements, allowing wide distributions in many countries. The pharmacological potential of the plants, as well as their safety and toxicity, has been critically studied in both in vitro and in vivo studies, and many studies have found compelling activities of *M. oleifera* as a potent agent with minimal toxicity. Hence, in this article, the phytochemical and pharmacological properties of *M. oleifera*, as well as the safety and efficacy, were comprehensively reviewed. The evidence of its antibacterial, anti-inflammatory, antiviral, anti-oxidative, cardio-protective, anti-diabetic, and anti-carcinogenic potentials was highlighted based on recent scientific publications. Despite its many potentials, the medicinal application of *M. oleifera* is still limited and not well advertised, especially based on the registered clinical trials for *M. oleifera*-related studies. Thus, as future research, more evaluation of the potential of *M. oleifera* should be carried out thoroughly for extensive data reviews prior to using it in clinical trials or human studies.

## Figures and Tables

**Table 1:** Summary of findings of safety and toxicity *of M. oleifera* in in vitro and in vivo studies.

Extracts	Concentration/Doses	Experimental Animal/Cell Line	Finding	Citation
In vitro studies				
Aqueous leaf extract	80.0, 40.0, 20.0, 10.0, 5.0 mg/mL of *M. oleifera* extract	Human peripheral blood mononuclear cells (PBMCs)	Lactate dehydrogenase (LDH) released upon cell damage or lysis indicates processes that occur during apoptosis and necrosis. Thus, the number of cells corresponds to the intracellular activity of LDH. As extracts increased above 20 mg/mL, the amount of LDH was released proportionally, indicating its cytotoxicity.	[9]
Aqueous seed extract	- Aqueous seed extract: 0.78–50 μg/mL - Diluted seed extract: 6.25–400 μg/mL (only for PBMCs)	PBMCs, human pulmonary mucoepidermoid carcinoma (NCI-H292), human colon adenocarcinoma (HT-29), human larynx epidermoid carcinoma (HEp-2)	The IC_50_ of 144 μg/mL for PBMCs treated with diluted seed extract (used to treat drinking water) indicates that it was not cytotoxic to PBMCs and had low cytotoxic activity toward other cancer cell lines. None of the evaluations showed hemolytic activity, indicating no damage to the plasma membrane of erythrocytes.	[10]
Aqueous (AM), methanolic (MM) and petroleum ether (EM) leaf extracts	5, 25 and 62.5 µg/mL	Human embryonic kidney cells expressing SV40 large T-antigen (293 T), Henrietta Lacks cells	The 50% cytotoxic concentration (TC_50_) was 41.58 µg AM/mL for aqueous extract, 38.88 µg MM/mL for methanolic extract and 32.33 µg EM/mL for ether extract in the assay performed by MTT assay.	[11]
Ethanolic leaf extracts	400 to 0.02 µg/mL (serial-fold dilution)	Fibroblast cell line	The cytotoxic concentration 50 (CC_50_) was at 100 µg/mL as cell survival percentage decreased to 50%. The CC_50_ for twelve concentrations (0.02 to 50 µg/mL) was lower and safe. Two concentrations (200 µg/mL and 400 µg/mL) were above CC_50_ and were cytotoxic to fibroblast cells.	[12]
Ethanolic seed extract	30, 50, 66, 83 and 100 µg/mL	Human colorectal carcinoma cells (HCT-116), Normal human cell lines (HEK-293)	Non-treated groups (cancer cells MOS treatment) showed 100% cell viability, whereas cancer cells treated with MOS extract showed a significant decrease, suggesting that the treatment led to significant drop in cancer cell viability. No inhibitory action observed in HEK-293 cells (non-cancerous cells), postulating that the extract is non-cytotoxic to normal cells.	[13]
Ethanolic leaf extracts	100 to 500 μg/mL	Human cervix carcinoma cell line (Hela)	Positive control significantly reduced the cell viability to less than 25%. Extracts were considered cytotoxic when they reduced cell viability to less than 50%. Leaf extract significantly and concentration-dependently reduced the viability of Hela cells at concentrations above 260 μg/mL.	[14]
In vivo studies				
Aqueous leaf extract	400, 800, 1600 and 2000 mg/kg	Rats (Male, Wistar rats)	2000 mg/kg dose showed no fatality, except decrease in body weight in a dose-dependent manner.	[15]
Aqueous seed extract	2000 mg/kg	Mice (Male, Balb/c)	No systemic toxicity, no significant changes in erythrocytes, platelets, hemoglobin and hematocrit observed.	[10]
Aqueous leaf extract	Low dose (1000 mg/mL), high dose (3000 mg/mL)	Rats (Male, Sprague Dawley)	*M. oleifera* shows acute toxicity at supra- supplementation levels of ≥3000 mg/kg bw.	[9]
Ethanolic leaf extracts	150 mg/mL dose every 5 min interval	White albino rats and rabbits (local breed)	Lethal dose for acute toxicity; 6616.67 mg/kg for rats and 26043.67 mg/kg for rabbits.	[16]
Aqueous-methanolic leaf extract	2000 mg/kg	Rats (Female, Wistar strain albino rats)	Increased levels of aspartate aminotransferase (AST) and total bilirubin. Decreased in alanine aminotransferase (ALT) level. Non-significant increase in hepatic index and mild distortions in liver cells via section analysis.	[17]
Crude methanol seed and leaf extracts	100, 200, 400 and 1000 mg/kg	Albino rats	No mortality at 1000 mg/kg dose. Seed extracts showed more potential in a long-term application.	[18]
Dried leaf powder	5, 50, 300 and 2000 mg/kg	Rats (Male and female, Sprague Dawley nulliparous and non-pregnant)	No adverse effect observed in clinical signs or gross pathology.	[19]

**Table 2:** Summary of studies on processing conditions, biological properties and phytochemical analyses of different parts of *M. oleifera* extracts.

Parts of the Plants	Extraction Solvent	Drying Method	Extraction Method	Analytical and Bioassay Approach	Phytochemical Content	Tested Biological Assay	Reference
Leaves	Leaf powder	Dried under the shade	NA	High-performance liquid chromatograph-photodiode array (HPLC-PDA)	Isoquercitrin (1494.2 µmol/100 g dried leaf powder) Rutin (1446.6 µmol/100 g dried leaf powder) Kaempferol glycosides (394.4 µmol/100 g dried leaf powder) Chlorogenic acid (134.5 µmol/100 g dried leaf powder) Quercetin (2030.9 µmol/100 g dried leaf powder) Kaempferol (336.7 µmol/100 g dried leaf powder)	Anti-diabetic (in vivo)	[39]
	Aqueous	NA	NA	Total phenolic content (TPC), 2,2-diphenyl-1-picryl-hydrazyl-hydrate (DPPH), ferric reducing antioxidant power assay (FRAP)	TPC (19.9 ± 2.5 *w*/*w* % gallic acid equivalents) DPPH (758.9 ± 23.8 IC_50_ mg/mL) FRAP (123.6 ± 7.1 ascorbic acid equivalents/mg dry plant)	Anti-glycating agent NPA: neuroprotective potential algorithm	[50]
		Air-dried	NA	NA	NA	Cardio-protective (in vivo)	[51]
		NA	Hot-water extraction	TPC, TFC, HPLC-UV	TPC (76.0 ± 2.4 μg/mg of extract) TFC (31.1 ± 4.1 μg/mg of extract) Chlorogenic acid (0.402 ± 0.04 μg/mg of extract) Caffeic acid (0.0133 ± 0.0096 μg/mg of extract)	Anti-oxidative (in vitro)	[52]
		Washed, dried and ground to powder using a mechanical grinder	Maceration	Standard phytochemical methods	Saponins (very high) Alkaloids (very high) Glycosides (very high) Tannins (abundant) Carbohydrates (moderate high) Acidic compounds (very high) Proteins (present)	Antiviral (in vitro)	[11]
		Air-dried under shade	12 h constantly shaken with 30 min intervals	NA	NA	Antibacterial (in vitro)	[53]
		Air-dried	Rotary shaker for 24 h	Standard phytochemical screening method	Tannin Phenol Flavonoid Saponins		[54]
		Dried in shade	Overnight infusion	NA	NA		[55]
		Air-dried	Maceration in water, 50 °C for 8 h				[56]
		NA	Intermittent shaking for 2 weeks				[57]
		NA	Cold-water extraction at 4 °C, vigorous and vortexes	NA	NA	Anti-carcinogenic (in vitro)	[58]
	Ethanol	Air-dried under shade	Soxhlet extraction in 80% ethanol, 24 h	NA	NA	Neuro-protective (in vitro and in vivo)	[59]
		Dried at 65 °C using a hot-air oven	Stirred for 8 h (ratio 1:4)	DPPH, FRAP	DPPH (58%) FRAP (26.76 ± 0.3 µmol/g)		[60]
		NA	Maceration with 80% ethanol at 70 ℃, 3 h	HPLC, TPC	Myricetin (5.44 mg 100/g) Kaempferol (1157.13 mg 100/g) TPC (69.75 μg/g)		[61]
		Dried under sun, pulverized by disintegrator	Ultrasonic extraction using water bath (200 W, 40 KHz) for 30 min in 90% ethanol	TFC LC-MS (HPLC-UV/ESI-MS/MS)	TFC (192.36 ± 2.96 mg rutin equivalent (RE)/g) Sucrose (RT 2.88) Niazirin (RT 2.95) Quinic acid isomer 2 (RT 191) Glucomoringin (RT 8.44) Acetyl-4-(α-l-rhamnopyranosyloxy) benzyl glucosinolate (RT 20.20) 3-Caffeoylquinic acid (RT 10.44) Rutin (RT 22.03) Quercetin 3-O-glucoside (RT 23.65) Quercetin-acetyl-glucoside (RT 26.47) Kaempferol 3-O-glucoside (RT 26.61) Kaempferol-acetyl-glycoside (RT 29.75)	Anti-oxidative (in vitro)	[62]
		NA	Soxhlet extraction	NA	NA	Antiviral (in vitro)	[63]
		Air-dried	Macerated for 72 h and stirred for 24 h in 95% ethanol	NA	NA	Antibacterial (in vitro)	[64]
		Air-dried under shade	12 h constantly shaken with 30 min intervals				[53]
		Air-dried	Rotary shaker for 24 h, 70% ethanol	Standard phytochemical screening method	Tannin Phenol Flavonoid Saponins		[54]
		Air-dried	Maceration in water, 50 °C for 8 h	NA	NA		[56]
		Vegetal tissue dehydrated using conventional oven for 3 days, 60 °C until constant weight	Stirred for 3 days in dark with ab. ethanol	Standard phytochemical methods	TPC (1356.57 ± 0.15 g/ mg GAE/100 g dry weight) TFC (1347.77 ± 0.13 QE mg/g dry weight)		[65]
		Air-dried at room temperature	Maceration with 98% ethanol, room temperature for 24 h	NA	NA	Anti-diabetic (in vivo)	[66]
		Dried in shade	Maceration with 95, 75, 50 and 25% (*v*/*v*) ethanol	HPLC-DAD	Saponin Tannin and Phenols Flavonoids Glycosides		[67]
		Air-dried	Maceration	NA	NA		[68]
		NA	Maceration for 6–8 h	GC-MS	Isopropyl isothiocyanate D-allose Cetene Palmitic acid	Anti-carcinogenic (in vitro and in vivo)	[69]
		Air-dried at room temperature for over 3 weeks	Stirred for 48 h	NA	NA		[70]
		NA	Ultrasound bath extraction (40 kHz, 300 W) for 1.5 h at 50 °C	TPC, TFC, FRAP	TPC (range from 27 ± 4 to 69 ± 4 mg GAE/g dry weight) TFC (range from 54 ± 3 to 115 ± 5 mg RE/g dry weight) FRAP (range from 160 ± 10 to 290 ± 5 mg/g)		[71]
	Methanol	Air-dried	Cold extraction	Standard phytochemical methods, HPLC-DAD and HPLC-EC, UV-visible spectroscopy (for vitamin content determination)	Alkaloid (low) Glycoside (low) Phenol (rich) Saponin (low) Tannin (low) Terpenoids (low) Flavonoid (very high, 22.6% *w*/*w*) Vitamin A (0.3 mg/g) Vitamin C (6.7 mg/g) Vitamin E (0.22 IU/g)	Neuro-protective (in vivo)	[72]
		NA	Maceration	Standard phytochemical screening method	Saponins (moderate high) Alkaloids (moderate high) Glycosides (very high) Tannins (very high) Carbohydrates (present) Flavonoid (moderate high) Resins (moderate high) Proteins (present)	Antiviral (in vitro)	[11]
		Air-dried under shade	12 h constantly shaken with 30 min intervals	NA	NA	Antibacterial (in vitro)	[53]
		Air-dried	Rotary shaker for 24 h, 80% methanol	Standard phytochemical screening method	Tannin Phenol Flavonoid Saponins		[54]
		Dried in shade	Soxhlet extraction for 3 h	NA	NA		[55]
		Air-dried at room temperature	Maceration	Standard phytochemical screening method	Alkaloids (++) Flavonoid (+++) Tannin (++) Phenol (+++)		[73]
		Air-dried	Soxhlet extraction for 8 h, in 80% methanol	NA	NA		[74]
	Chloroform	NA	Intermittent shaking for 2 weeks	NA	NA	Antibacterial (in vitro)	[57]
	Ethyl acetate						
	Butanol						
	Petroleum ether	NA	Maceration	Standard phytochemical methods	Alkaloids (abundant) Flavonoid (abundant) Resins (present)	Antiviral (in vitro)	[11]
Flower/pulp	Aqueous	Air-dried	Rotary shaker for 24 h	Standard phytochemical screening method	Flower Flavonoid Saponins Pulp Tannin Phenol Saponins	Antibacterial (in vitro)	[54]
	Ethanol		Rotary shaker for 24 h, 70% ethanol		Tannin Phenol Saponins		
	Methanol		Rotary shaker for 24 h, 80% methanol		Flower Tannin Phenol Pulp 3. Tannin 4. Phenol 5. Saponins		
	Petroleum ether		Rotary shaker for 24 h		Flower Saponins Pulp 2. Tannin 3. Phenol 4. Saponins		
Stem	Aqueous	Dried in shade	Maceration for 72 h, room temperature	NA	NA	Antibacterial (in vitro)	[75]
		NA	Hot-water extraction	TPC, TFC, HPLC-UV	TPC (61.5 ± 1.8 μg/mg of extract) TFC (6.89 ± 1.54 μg/mg of extract) Chlorogenic acid (0.120 ± 0.001 μg/mg of extract) Caffeic acid (0.00571 ± 0.00275 μg/mg of extract)	Anti-oxidative (in vitro)	[52]
	Ethanol	NA	Ultrasound bath extraction (40 kHz, 300 W) for 1.5 h at 50 °C	NA	NA	Anti-carcinogenic (in vitro)	[71]
	Methanol	Dried in shade	Maceration for 72 h in 99.9% methanol, room temperature	NA	NA	Antibacterial (in vitro)	[75]
	n-Hexane		Maceration for 72 h in 98.9% n-hexane, room temperature				
Bark	Ethanol	NA	Maceration for 6–8 h	GC-MS	Eugenol Dibutyl phthalate 2- chloropropionic acid 5-eicosene Palmitic acid	Anti-carcinogenic (in vitro)	[69]
Root	Ethanol	Dried under sun, pulverized by disintegrator	Ultrasonic extraction using water bath (200 W, 40 KHz) for 30 min in 90% ethanol	TFC LC-MS (HPLC-UV/ESI-MS/MS)	TFC (106.79 ± 2.12 mg rutin equivalent (RE)/g) Sucrose (RT 2.88) Cellotriose (RT 2.90) Methyl 4-caffeoylquinate (RT 2.87) Quinic acid isomer (RT 3.20) Glucomoringin (RT 8.44) Glucotropaeolin (RT 16.90)	Anti-oxidative (in vitro)	[62]
		NA	Ultrasound bath extraction (40 kHz, 300 W) for 1.5 h at 50 °C	NA	NA	Anti-carcinogenic (in vitro)	[71]
Seed	Seed powder	Dried and ground to powder	NA	NA	NA	Cardio-protective (in vivo)	[76,77,78]
		Dried in shade				Anti-oxidative (in vivo)	[79]
	Seed oil	Air-dried in oven	Soxhlet extraction using hexane, heated at low temperature	NA	NA	Anti-diabetic (in vivo)	[80]
	Aqueous	Dried in shade	Stirred for 48 h, room temperature	TPC, TFC, Total tannin	TPC (90.97 ± 0.134 mg GAE/g of dry extract) TFC (221.76 ± 0.221 mg QE/g of dry extract) Total tannin (21.74 ± 0.086 mg GAE/g of dry extract)	Anti-oxidative (bioassays)	[81]
		Dried in shade	Maceration for 72 h, room temperature	NA	NA	Antibacterial (in vitro)	[75]
			Rotary shaker for 2 days, cold and hot water				[82]
		Air-dried for 2 days	Shaking on horizontal shaker for 3 days				[83]
		NA	Maceration for 72 h at room temperature	NA	NA	Anti-diabetic (in vivo)	[80]
	Ethanol	Dried under sun, pulverized by disintegrator	Ultrasonic extraction using water bath (200 W, 40 KHz) for 30 min in 90% ethanol	TFC LC-MS (HPLC-UV/ESI-MS/MS)	TFC (5.89 ± 0.65 mg rutin equivalent (RE)/g) Cellotetraose (RT 2.83) Sucrose (RT 2.88) Cellotriose (RT 2.90) 3-Hydroxy-4-(α-l-rhamnopyranosyl oxy) benzyl glucosinolate (RT 7.94) Glucomoringin (RT 8.44)	Anti-oxidative (in vitro)	[62]
		Dried in shade	Rotary shaker for 2 days	NA	NA	Antibacterial (in vitro)	[82]
		NA	Ultrasound bath extraction (40 kHz, 300 W) for 1.5 h at 50 °C	NA	NA	Anti-carcinogenic (in vitro)	[71]
		NA	Maceration for 6–8 h	GC-MS	1-butanamine 1-dodecene 2-decenal 3-tetradecene 2-tetradecene 1-octadecene hexadecanoic acid 10-octadecenoic acid heptadecanoic acid		[69]
		NA	Maceration in 70% ethanol at 85 °C, 2 h	NA	NA	Neuro-protective (in vivo)	[84]
	Methanol	Dried in shade	Soxhlet extraction	Standard phytochemical screening method	Flavonoids Tannins Glycosides Carbohydrates	Cardio-protective (in vivo)	[85]
		Dried in shade	Stirred for 48 h, room temperature	TPC, TFC, Total tannin	TPC (60.99 ± 0.153 mg GAE/g of dry extract) TFC (10.13 ± 0.171 mg QE/g of dry extract) Total tannin (97.10 ± 0.153 mg GAE/g of dry extract)	Anti-oxidative (bioassays)	[81]
		De-shelled, dried at 23 to 25 °C for 5 days	Shaken for 4 h and macerated overnight	NA	NA	Antibacterial (in vitro)	[86]
		Dried in shade	Maceration for 72 h in 99.9% methanol, room temperature				[75]
			Rotary shaker for 2 days				[82]
		Air-dried for 2 days	Shaking on horizontal shaker for 3 days				[83]
		Air-dried	Soxhlet extraction for 8 h				[74]
	Acetone	Dried in shade	Stirred for 48 h, room temperature	TPC, TFC, Total tannin	TPC (30.78 ± 0.101 mg GAE/g of dry extract) TFC (13.32 ± 0.101 mg QE/g of dry extract) Total tannin (73.91 ± 0.107 mg GAE/g of dry extract)	Anti-oxidative (bioassays)	[81]
		Air-dried for 2 days	Shaking on horizontal shaker for 3 days	NA	NA	Antibacterial (in vitro)	[83]
	Petroleum ether	Air-dried	Soxhlet extraction for 4 h, 40–60 °C				[74]
	Hexane	Dried in shade	Maceration for 72 h in 98.9% n-hexane, room temperature				[75]

**Table 3:** Extracts of *M. oleifera* and the extensive findings on antibacterial properties.

Extracts	Application	Finding	Citation
70% Ethanol, 80% methanol, petroleum ether and aqueous extracts of *M. oleifera* leaves, flower, pulp and seed	Method: Agar-well diffusion method Bacteria species: *E. coli* and *S. aureus*	Maximum zone of inhibition: Leaves: 80% methanol extract against *E. coli* (28 mm) and *S. aureus* (26 mm). Flower: 70% ethanol extract against *E. coli* (23 mm) and *S. aureus* (17 mm). Pulp: 80% methanol extract against *E. coli* (15.33 mm). Aqueous extracts against *S. aureus* (18.33 mm). Seed: 80% methanol extract against *E. coli* (18.33 mm). 70% ethanol extracts against *S. aereus* (15.66 mm).	[54]
Aqueous, petroleum ether and methanolic (20, 40, 60%) extracts of *M. oleifera* leaf	Method: In vitro, cup–plate method and disc diffusion method Bacteria species: *S. aureus*, *E. coli*, *Klebsiella pneumonia*, *P. aeruginosa* and *Proteus vulgaris*	Methanolic extracts (20, 40, 60%) had high inhibitory effects on *S. aureus*, *K. pneumoniae* standard strains and *S. aureus*, *S. saprophyticus* and *E.coli* isolated from urinary tract infection. Aqueous extract only showed effects on *P. vulgaris* standard strain. Petroleum ether extracts showed no inhibitory activity at all.	[55]
Methanol (99.9%), n-hexane (98.9%) and aqueous extracts of *M. oleifera* and *M. ovalifolia* seeds and bark	Method: Paper-disc diffusion method Bacteria species: *E. coli*, *Enterococcus faecalis* and *Bacillus cereus*	*M. oleifera* extracts showed higher inhibitory activities than *M. ovolifolia*. Seed extracts of *M. oleifera* exhibit a wider range of antibacterial activity than *M. ovalifolia.* *M. oleifera* bark extracts showed higher antibacterial activity than *M. ovalifolia* against all tested species. n-hexane extracts for both *M. ovalifolia* and *M. oleifera* showed similar inhibitory activities but were generally lower than other solvents.	[75]
Aqueous and ethanolic *M. oleifera* leaf	Method: Agar diffusion and microbroth dilution methods Bacteria species: *S. aureus*, *Streptococcus pyogenes*, *Bacillus cereus*, *E. coli*, *P. aeruginosa*, *Shigella sonnei*, *Shigella dysenteriae*, *Shigella flexneri*, *Shigella boydii* and *Proteus mirabilis*	All tested bacterial isolates were observed to be susceptible to both extracts at 100 µg concentration, but susceptibility decreased as the extract concentration reduced. *M.oleifera* leaf extract showed broad spectrum of antibacterial activities as it works on both Gram-positive and -negative bacteria. Ethanol extract exhibited higher inhibition and minimal inhibitory concentrations.	[56]
Chloroform, ethyl acetate, butanol and aqueous extracts *M. oleifera* leaf	Method: In vitro, agar-well diffusion method Bacteria species: *E. coli*, *P. vulgaris*, *K. pneumoniae*, *Salmonella enterica*, *P. aeruginosa*, *S. aureus*, *Staphylococcus epidermidis* and *B. cereus*	Ethyl acetate extract observed the highest antibacterial activity against *S. epidermidis*, *S. aureus*, *P. aeruginosa* and *B. cereus.* Butanol extract reacted against *S. epidermidis* and *S. aureus*. Aqueous and chloroform extract only showed activity against *S. epidermidis* and *S. aureus*, respectively.	[57]
Methanolic *M. oleifera* leaf	Method: In vitro, agar-well diffusion method Bacteria species: *E. coli* and *Klebsiella*	The extracts showed activities against both bacteria in dose-dependent manner, as such highest activities were observed at dose of 200 mg/L. Methanol extract of *M. oleifera* was shown to have minimum inhibitory concentration (MIC) value against Kleibsilla at 45 mg %.	[73]
Aqueous (hot and cold), ethanolic and methanolic extracts of *M. oleifera* seeds	Method: In vitro, disc diffusion method and broth dilution method Bacteria species: *S. aureus*, *E. coli* and *P. aeruginosa*	The MIC for different extracts was observed as follows: aqueous (cold) extract showed no MIC, but aqueous (hot) extract was 100 mg/mL. The MIC for ethanolic and methanolic extracts is also capped at 100 mg/mL, except for *E. coli* and *S. aureus* with MIC of 50 mg/mL. The minimum bactericidal concentration (MBC) of aqueous (hot), ethanolic and methanolic extracts on tested bacteria was 200 mg/mL, but methanolic extract showed MBC of 100 mg/mL on *E. coli*.	[82]
Methanol, acetone and aqueous extracts of *M. oleifera* seeds	Method: In vitro, agar-well diffusion technique and MIC and MBC Bacteria species: *E. coli*, *Shigella typhii* and *Shigella dysenteriae*	Acetone extracts showed highest antibacterial activity against *S. typhii* and least sensitivity with the aqueous extract. The most observed MIC value was 6.25 mg/mL, then 12.5 mg/mL. Acetone extract is the most potent in exhibiting inhibitory activities at very low concentration for *Shigella typhii*	[83]
80% methanolic extracts of *M. oleifera* leaf and seeds	Method: In vitro, agar-well diffusion method Bacteria species: *E. coli*, *s. typhi*, *salmonella paratyphi- A*, *salmonella paratyphi-B*, *shigella dysentriae*, *s. aureus*, *streptococcus feacalis*, *p. aeruginosa*, *Proteus mirabilis* and *k. Pneumoniae*	Both leaf and seed extracts exhibit antibacterial activity against all bacterial ATCC strains, but for leaf extract, highest activity was observed on *S. typhi* (ATCC19430) while the seed extracts showed on *E. coli* (ATCC25922). Both extracts also showed activities in clinically isolated bacterial strains, but for leaf extract, highest activity was observed against *S. aureus*, and for seed extract, highest activities was observed against *K. pneumoniae*, *P. mirabilis* and *S. typhi.* Overall, the results show higher antibacterial activity in leaf extract as compared to seed extract.	[74]
Ethanolic extracts of *M. oleifera* leaf	Method: Bacteria inhibition microplate assay Bacteria species: *Agrobacterium tumefeciens (At)*, *Clavibacter michiganensis subsp. michiganensis (Cmm)*, *Pseudomonas syringae pv. tomato (Pst)*, *Ralstonia solanacearum (Rs)* and *Xanthomonas axonopodis (Xa)*	Results show that the extract exhibits inhibitory effects on the growth of phytopathogenic bacteria *At*, *Cmm*, *Pst*, *Rs* and *Xa* in dose-dependent manner. Higher inhibition was observed at higher concentration of extract. *At* was found to be most susceptible to the exact treatment while *Rs* was more resistant. Ethanolic extracts of *M. oleifera* leaf showed prominent bio-bactericide potential.	[65]

**Table 4:** The overview of all registered clinical trials of *M. oleifera*.

Status	Official Title	Intervention/ Treatment	Outcome Description	Study Design
Completed Duration: December 2012–August 2014	Safety and efficacy of Chandrakanthi Choornam in Oligospermia—A preclinical and clinical study	Drug: Chandrakanthi Choornam (CKC)—formulation consisting of 25 ingredients Conditions: Oligospermia	Determination of sperm concentration, motility, and proportion of sperm morphology, as well as the effects on hormonal level.	Primary Purpose: Treatment Allocation: N/A Interventional Model: Single Group Assignment Masking: None (Open Label)
Completed Duration: January 2013–September 2013	Effect of *M. oleifera* (Moringa, Drumstick/Horseradish Tree) on the pharmacokinetics of Efavirenz and Nevirapine in-vivo.	Dietary Supplement: *M. oleifera* Drug: Efavirenz (600 mg) and Nevirapine (200 mg) *Oral Tablet Conditions: HIV	Pharmacokinetic endpoints include area under the curve (AUC), time to maximum plasma concentration (tmax), peak plasma concentration (Cmax), trough concentration (Cmin), clearance (CL), volume of distribution (Vd/F) and half-life (T1/2).	Observational Model: Case-Crossover Time Perspective: Prospective Biospecimen Retention: SAMPLES_WITH_DNA Biospeciman Description: Whole Blood, Plasma, Urine
Completed Duration: January 2013–April 2014	Impact of dried *M. oleifera* leaves as value added supplement in enhancing hemoglobin status of reproductive aged females of low socio-economic group	Dietary Supplement: Dried *M. oleifera* leaves Conditions: Anemia, iron deficiency	Improvement in hemoglobin status and body mass index.	Primary Purpose: Supportive Care Allocation: Non-Randomized Interventional Model: Single Group Assignment Masking: None (Open Label)
Completed Duration: August 2014–November 2014	The effects of *M. oleifera* supplements on hsCRP and HgbA1c levels of patients in Hospital Ng Maynila Medical Center diabetic clinic: A prospective cohort study	Dietary Supplement: *M. oleifera* Conditions: Diabetes	Post treatment means of inflammatory markers HsCRP and hgba1c.	Primary Purpose: Supportive Care Allocation: N/A Interventional Model: Single Group Assignment Masking: None (Open Label)
Completed Duration: January 2014–April 2022	An observational clinical study to determine the effect of multi-modal Ayurvedic treatment in the patients of chronic kidney disease	Other: Ayurveda treatment Conditions: Diabetic nephropathy, hypertensive nephropathy	The determination of the signs and symptoms as well as the improvement in metabolic profiles.	Primary Purpose: Treatment Allocation: N/A Interventional Model: Single Group Assignment Masking: None (Open Label)
Completed Duration: July 2015–May 2016	Effect of *M. oleifera* on bone density in post-menopausal Women	Dietary Supplement: *M. oleifera* Dietary Supplement: Cabbage placebo Conditions: Osteoporosis, osteopenia, postmenopausal, osteoporosis	Bone density determined by dual-energy X-ray absorptiometry.	Primary Purpose: Basic Science Allocation: Randomized Interventional Model: Parallel Assignment Masking: Double
Completed Duration: March 2016–April 2016	Evaluation of the effect of *M. oleifera* tea on metformin steady state plasma level in type 2 diabetes mellitus patients—A pre and post non-randomised trial	Dietary Supplement: *M. oleifera* tea Conditions: Type 2 diabetes mellitus	Changes in fasting blood glucose, 2 h post prandial blood glucose, metformin plasma concentration, estimated glomerular filtration rate.	Primary Purpose: Treatment Allocation: N/A Interventional Model: Single Group Assignment Masking: None (Open Label)
Completed Duration: November 2016–May 2017	Short term cardiovascular and renal effects of *M. oleifera* extracts and Stevia *Rebaudiana Bertoni* as add-on therapy in a population of type II diabetes individuals	Dietary Supplement: MOROLSTEVER1 (Capsules of *M. oleifera* and Stevia *Rebaudiana Bertoni*) Conditions: Type 2 diabetes mellitus	Determination of diastolic function, early diastolic filling velocity, urinary excretion of albumin and changes in blood pressure.	Primary Purpose: Treatment Allocation: N/A Interventional Model: Single Group Assignment Masking: None (Open Label)
Completed Duration: December 2017–August 2020	Evaluation of the effect of *Artemisia annua* and *M. oleifera* on immunological response in HAART HIV patients	Dietary Supplement: *Artemisia Annua*, *M. oleifera* Conditions: HIV infections	Determination of CD4 counts, viral load, complete blood count, immunoglobins, antiretroviral plasma drug level, patients’ perceptions on mental and physical quality of life, liver function biomarkers, side effects or adverse drug reactions, renal function biomarkers.	Primary Purpose: Treatment Allocation: Randomized Interventional Model: Parallel Assignment Masking: Double
Completed Duration: September 2018–March 2019	MORINGA; Delivering nutrition and economic value to the people of Malawi	Other: Control corn soya diet Other: Test corn, moringa diet Conditions: Malnourishment	Determination of phytochemical metabolite concentration in systemic circulation.	Primary Purpose: Prevention Allocation: Randomized Interventional Model: Crossover Assignment Masking: None (Open Label)
Completed Duration: January 2019–January 2021	Effects of *M. oleifera* Leaves on glycemia, lipemia and inflammatory profile: Nutritional intervention study in prediabetic patients	Dietary Supplement: Moringa Dietary Supplement: Placebo Conditions: Pre-diabetes	Determination of fasting blood glucose and glycated hemoglobin (HbA1C), conversion rate from prediabetes to normal, concentration of total serum cholesterol, lipoprotein–cholesterol, inflammatory markers and metabolic hormones, antioxidant capacity and microbiota composition.	Primary Purpose: Prevention Allocation: Randomized Interventional Model: Parallel Assignment Masking: Quadruple
Completed Duration: January 2020–March 2021	An evaluation of the effects of a non-caffeinated energy dietary supplement on cognitive and physical performance: A randomized double-blind placebo-controlled study	Dietary Supplement: Phytovive, placebo, caffeine Conditions: Health behavior	Evaluation of cognition, mood and physical performance.	Primary Purpose: Basic Science Allocation: Randomized Interventional Model: Parallel Assignment Masking: Double
Unknown status Duration: February 2020–July 2020	Effect of aerobic training and *M. oleifera* on dyslipidemia and cardiac endurance	Other: Aerobic training and *M. oleifera* Conditions: Dyslipidemias	Change in level of HDL, LDL and triglycerides, impact on cardiac endurance.	Primary Purpose: Health Services Research Allocation: Randomized Interventional Model: Parallel Assignment Masking: None (Open Label)
Unknown status Duration: February 2020–August 2020	Randomized clinical study investigating the effect of *M. oleifera* infusion on bioclinical parameters of health	Dietary Supplement: *M. oleifera* tea Conditions: Metabolic syndrome	Change in blood glucose level, LDL cholesterol and triglyceride level as well as antioxidants of blood (e.g., superoxide dismutase, glutathione peroxidase, total blood antioxidant capacity).	Primary Purpose: Other Allocation: Randomized Interventional Model: Crossover Assignment Masking: Single
Completed Duration: March 2021–June 2022	Effect of *M. oleifera* capsule in increasing breast milk volume in early postpartum patients: A double blind randomized controlled trial	Drug: *M. oleifera* leaf Drug: Placebo Conditions: Postpartum women	Determination of breast milk volume at postpartum day 3, as well as percentage of good satisfaction, quality of life scores, side effects and compliance.	Primary Purpose: Other Allocation: Randomized Interventional Model: Parallel Assignment Masking: Quadruple
Completed Duration: July 2021–November 2021	Investigating the impact of *M. oleifera* leaf supplementation on growth, nutrition, lactation, and inflammation in Kenyan breastfeeding mothers and children	Dietary Supplement: *M. oleifera* (high dose and low dose) Dietary Supplement: Placebo Conditions: Malnutrition, wasting, growth failure	Changes in physical descriptions (body weight, height, mid-upper arm and head circumference) and metabolite levels (vitamin A, C-reactive protein, soluble transferrin–ferritin index, fecal neopterin, fecal myeloperoxidase, alpha-1-antitrypsin). The prevalence of diarrhea and changes in breast milk (output, vitamin A, retinol, vitamin E, catalase, lactoferrin) were also determined.	Primary Purpose: Prevention Allocation: Randomized Interventional Model: Single Group Assignment Masking: Single
Active, not recruiting Duration: December 2021–October 2022	Effects of *M. oleifera* mouthwash in patients undergoing fixed orthodontic appliance treatment: A parallel arm, triple blinded, randomized controlled trial	Other: *M. oleifera* mouthwash Other: Placebo mouthwash Conditions: Gingivitis, periodontitis, plaque formation, enamel demineralization/white spot lesion, discoloration and bacterial load in dental plaque (orthodontic appliance complication)	Periodontal probing depth, plaque index, white spot lesions, modified gingival index, discoloration of teeth, bacterial load in plaque.	Primary Purpose: Prevention Allocation: Randomized Interventional Model: Parallel Assignment Masking: Quadruple
Completed Duration: February 2021– December 2021	Antifungal potential of *M. oleifera*-loaded nanoparticles against otomycosis; Preparation, characterization, and Clinical Evaluation	Drug: *M. oleifera* leaf 10 mg/100 mL Drug: Ear drop Conditions: Otomycosis	The number of participants recovered with clear endoscopic examination and identification of different microorganisms that infected ear were determined.	Primary Purpose: Treatment Allocation: Randomized Interventional Model: Parallel Assignment Masking: None (Open Label)
Active, not recruiting Duration: April 2021–February 2022	Child health, agriculture, and integrated nutrition (CHAIN): A randomized trial to close the nutrient gap in rural Zimbabwe	Dietary Supplement: Small-quantity lipid-based nutrient supplement, provitamin A biofortified maize, NUA-45 biofortified sugar beans, *M. oleifera*, whole egg powder and white maize meal Conditions: Stunting	Determinations of protein, iron, zinc, folate and energy intake, height and weight for age score, hemoglobin level, microbiome maturity, environmental enteric dysfunction, innate immune cell phenotype and function, plasma essential amino acids and choline, as well as urinary metabolic secretion.	Primary Purpose: Prevention Allocation: Randomized Interventional Model: Parallel Assignment Masking: Single
Recruiting Duration: August 2021–March 2022	A randomized, double-blind, cross-over, placebo-controlled study to explore the effect of *M. oleifera* (E-HS-01) on flow mediated dilatation and hemodynamics	Other: *M. oleifera* (E-HS-01) Other: Placebo Conditions: Endothelial function	Determination of flow-mediated dilatation and blood flow velocity.	Primary Purpose: Other Allocation: Randomized Interventional Model: Crossover Assignment Masking: Quadruple
Recruiting Duration: April 2022–July 2022	Evaluation of the anti-plaque and anti-gingivitis effects of Moringa plant extract and fluoride toothpastes among a group of Egyptian children: A randomized clinical trial	Drug: *M. oleifera* leaf Conditions: Oral disease	Determination of gingival index and plaque index.	Primary Purpose: Treatment Allocation: Randomized Interventional Model: Parallel Assignment Masking: Double
Recruiting Duration: May 2022–May 2023	Investigating the effect of *M. oleifera* leaf powder on breastmilk quantity and quality: A double blinded randomized placebo-controlled trial	Dietary Supplement: Moringa leaf powder Other: Placebo Conditions: Breastfeeding	Differences in milk output, species prevalence of infant microbiome and maternal milk microbiome, maternal milk fat and protein, infant fecal and maternal milk microbiome composition, maternal milk vitamin A.	Primary Purpose: Treatment Allocation: Randomized Interventional Model: Parallel Assignment Masking: Quadruple
Not yet recruiting Duration: July 2022- December 2023	Antibacterial, antiplaque and anticariogenic effect of *M. oleifera* mouthwash compared to chlorhexidine mouthwash: A randomized clinical trial	Drug: *M. oleifera*, chlorhexidine mouthwash, base formula Conditions: Plaque, dental antimicrobial, mouthwash cytotoxicity	Determinations of gingival index (GI) and white spot lesions.	Primary Purpose: Prevention Allocation: Randomized Interventional Model: Parallel Assignment Masking: Double
Not yet recruiting Duration: August 2022–April 2023	Effect of *M. oleifera* leaf extract versus sodium hypochlorite as root canal irrigant on postoperative pain and bacterial reduction in mandibular premolars with necrotic pulps: A randomized clinical trial	Dietary Supplement: *M. oleifera* leaf Drug: Sodium hypochlorite Conditions: Necrotic pulp	Determinations of postoperative pain and intracanal bacterial load change.	Primary Purpose: Treatment Allocation: Randomized Interventional Model: Parallel Assignment Masking: Double
